# Characterising Informal Care in Older Individuals Receiving Long‐Term Home Care Support: A National Epidemiological Study

**DOI:** 10.1111/ajag.70128

**Published:** 2026-01-22

**Authors:** Tesfahun C. Eshetie, Alana R. Cuthbert, Janet K. Sluggett, Solomon Yu, Carolyn Dawkins, Marjorie Schulze, Gillian E. Caughey, Maria C. Inacio

**Affiliations:** ^1^ Registry of Senior Australians Research Centre South Australian Health and Medical Research Institute Adelaide South Australia Australia; ^2^ Registry of Senior Australians Research Centre, Caring Futures Institute, College of Nursing and Health Sciences Flinders University Bedford Park South Australia Australia; ^3^ University of South Australia UniSA Allied Health and Human Performance Adelaide South Australia Australia; ^4^ University of South Australia UniSA Clinical and Health Sciences Adelaide South Australia Australia; ^5^ Adelaide Geriatrics Training and Research With Aged Care (GTRAC) Centre, Faculty of Health and Medical Sciences, Adelaide Medical School University of Adelaide Adelaide South Australia Australia; ^6^ Aged and Extended Care Services, The Queen Elizabeth Hospital and Basil Hetzel Institute for Translational Research Central Adelaide Local Health Network, SA Health Adelaide South Australia Australia; ^7^ ECH Inc Parkside South Australia Australia

**Keywords:** dementia, home care, informal care, informal caregivers, long‐term care

## Abstract

**Objective:**

To examine the prevalence, trends and geographic variation of informal care reported by individuals accessing long‐term home care support between 2012 and 2019 in Australia.

**Methods:**

Population‐based national cross‐sectional study using the Registry of Senior Australians (ROSA) National Historical cohort. Non‐Indigenous individuals 65–105 years old who accessed long‐term home care through a Home Care Package between 01 January 2012 and 31 December 2019 in Australia were included. Informal carer availability was ascertained from individuals' aged care eligibility assessments. Informal carers are individuals who provide unpaid care and support to others. Socio‐demographic and clinical characteristics of those with and without informal carers were examined. Yearly trends and geographic variation in the proportion of individuals reporting a carer were examined. The effect of a 1‐year increase in receiving initial long‐term home care on the probability of having a carer over time was described using an odds ratio (OR) and 95% confidence interval (95% CI) from a logistic regression model, adjusted for age, sex and dementia status.

**Results:**

Overall, 233,567 long‐term home care recipients with known carer status were studied. The proportion of care recipients with an informal carer decreased from 86% in 2012 to 78% in 2019 (adjusted OR: 0.95, 95% CI 0.95–0.95). The decrease in informal care reported over time was more pronounced in females (OR: 0.96, 95% CI 0.95–0.97) than in males and in individuals without dementia (OR: 0.95, 95% CI 0.94–0.95). Visualisations of informal care prevalence showed substantial geographical (range: 60%–98%) variation nationally.

**Conclusions:**

There was a decline in reported informal care availability for older Australians entering long‐term home care between 2012 and 2019, with substantial national variation. Lower informal carer availability likely translates in greater formal care needs.

## Abstract (highlights)

**Practice Impact:**

Our study found a decline in reported informal carer availability for older people in long‐term home care during the study period, highlighting the importance of strengthened informal care networks to support safe and successful ageing in place.

**Policy Impact:**

Substantial geographic disparities in reported informal carer availability for older people in long‐term home care across Australia, particularly lower rates in socio‐economically disadvantaged areas, remote regions and metropolitan city centres, highlight the need for targeted policy interventions and support systems to empower informal carers.

## 1 Introduction

The contribution of informal carers, who are individuals providing unpaid care and support to others, is substantial in supporting older people, with an estimated 13% of the population over 50 years old in 23 Organisation for Economic Co‐operation and Development (OECD) countries providing informal care at least weekly on average [1]. In Australia, as common in other countries [1], health care and social services sectors (i.e., aged care and disability) are dependent on informal carers to support older people [2]. According to the 2022 Survey of Disability, Ageing and Carers conducted by the Australian Bureau of Statistics, 3 million Australians provided informal care in 2022 (~12% of the population), an increase from 2.6 million in 2018, with 1.2 million identified as primary carers [3]. As Australia's population continues to age, the demand for informal care is estimated to grow by 23% by 2030 (1.3 million in 2020 to 1.54 million in 2030) across all age groups with a severe restriction living in the community [2].

Across most OECD countries, there has been a growing trend towards delivering long‐term aged care at home rather than in institutional settings, driven by policies prioritising ageing in place [4]. In an effort to support older individuals to live at home for as long as they wish, the Australian Government has provided support for long‐term home care to older people since 1985 [5]. Since 2013, this program, which is now known as the Home Care Packages Program, has increased in size dramatically and in 2023 315,000 people were accessing the services, which is more individuals than in residential care settings [6]. This program offers bundled personal care, support services, nursing, allied health or clinical services (determined by an individual's needs) to individuals through a four‐level system of packages, ranging from basic care (Level 1) to high care (Level 4) support [7]. However, delivering high‐quality care to older Australians in this setting has been a challenge nationally, as highlighted by the Royal Commission into Aged Care Quality and Safety (2018–2021) and studies that suggest that individuals in long‐term home care are more often hospitalised than residents of long‐term care facilities [8, 9, 10], particularly for certain events like weight loss/malnutrition, medication‐related events and delirium or dementia‐related hospitalisation. Carers are a critical facilitator of individuals being able to live at home longer and contribute to their experiences while ageing at home.

The Australian Government has acknowledged the importance of informal carers and recently released a National Carer Strategy 2024–2034 to provide a national framework to improve the lives of Australia's unpaid carers [11]. Additionally, in response to the Aged Care Royal Commission recommendations, the government invested $798.3 million in 2021–2022 to increase support provided for informal and family carers of older Australians, particularly for those caring for people living with dementia, but the effect of such reforms is still to be evaluated [8].

Past research on informal care for older Australians has largely drawn from national surveys, such as the Household, Income and Labour Dynamics in Australia survey [12] and the Survey of Disability, Ageing and Carers [13]. However, there is limited evidence on the characteristics and access to informal care among older individuals receiving long‐term home care, as well as their caregivers' experiences. Significant gaps exist in understanding informal care availability for priority populations, such as rural/remote and culturally diverse communities [11, 14]. It is unclear that the findings from existing population‐wide longitudinal surveys are generalisable to long‐term home care recipients [12, 15]. Additionally, limited evidence exists on the national impact of long‐term home care recipients' informal care burden, the sustainability of caring relationships and the extent to which care recipients are acting as a carer themselves. A comprehensive understanding of carer‐recipient dyads at the population level is therefore needed to better inform policies and practices of long‐term in‐home aged care. To address these gaps, we examined the prevalence, trends and geographic variation of informal care reported by individuals accessing long‐term home care support between 2012 and 2019 in Australia.

## 2 Methods

### 2.1 Study Design, Data Sources and Settings

A population‐based cross‐sectional study was undertaken using the ROSA National Historical cohort. Briefly, the ROSA National Historical Cohort integrates deidentified national and state‐based datasets from aged care, health care and social welfare sectors for older people who have undergone an aged care eligibility assessment or accessed aged care services from 2002 onwards, and has been described in detail elsewhere [16]. This study includes the following datasets contained in the ROSA: Australian Institute of Health and Welfare's National Aged Care Data Clearinghouse (NACDC) data collections (which includes the National Death Index, NDI), Australian Government Data On Multiple INdividual Occurrences (DOMINO), Medicare Benefits Schedule and Pharmaceutical Benefits Scheme. The NACDC contains the aged care eligibility assessments, collected through either an Aged Care Assessment Program (ACAP, 2013–2016) or National Screening and Assessment Form (NSAF, 2016–2019), of individuals entering the aged care sector and the episodes of care delivered by aged care providers.

### 2.2 Study Cohort

All non‐Indigenous individuals 65–105 years old who initiated long‐term home care through a Home Care Package between 01 January 2012 and 31 December 2019 in Australia were included (Figure S1).

### 2.3 Measures

The outcome of interest was informal care availability (hereafter referred to as ‘carers’) reported by long‐term home care recipients during their most recent aged care eligibility assessment (i.e., ACAP and NSAF) prior to the date of receiving their first long‐term home care, which included (i) availability and (ii) specific carer support provided. In these assessment tools, an informal carer is someone (a family member, friend or neighbour, excluding paid or volunteer carers organised by formal services) who provides care and support to a person without payment other than a pension or benefit.

In 2016, the NSAF replaced the ACAP as the tool for assessing eligibility for Australian government‐funded aged care services (Figure S2). If a care recipient had multiple aged care eligibility assessments before receiving their first home‐based long‐term care, then the most recent assessment was used. Although carer availability was consistently collected in both ACAP and NSAF assessment tools during the study period, the carer information available in these tools varied (Tables S2 and S3, Figures S2 and S3, Methods S1). Specifically, only the ACAP (~2013–2016) included carers' co‐residency status, relationship and respite care use and only the NSAF (~2016–2019) included family and other support networks, social and community participation, change in caring arrangement, home care recipient as a carer and sustainability of caring relationships [17, 18].

Variables used to characterise individuals in this study included socio‐demographic and clinical characteristics, medicine use and health care utilisation‐related characteristics (Tables S4–S7). Socio‐demographic and clinical characteristics were ascertained during the aged care eligibility assessment, including age, sex (male/female), country of birth, living arrangements, Australian state or territory, remoteness, culturally and linguistically or ethnically diverse status, preferred language (English vs. other), Socio‐economic Indexes for Areas (SEIFA) Index of Relative Socio‐economic Advantage and Disadvantage [19], number of comorbidities (pharmaceutical‐based comorbidity index [20]), and individual health conditions focusing on geriatric syndromes and selected medical conditions that may require an informal carer [21] (i.e., dementia, history of falls, history of fractures, history of pressure injury, incontinence, history of delirium, epilepsy, Parkinson's disease, stroke/cerebrovascular disease, diabetes, cancer, ischaemic heart disease, blindness and deafness). Medicine use prior to entry to long‐term home care was ascertained from the Pharmaceutical Benefits Scheme dispensing records. This included the number of unique medications dispensed, antipsychotic use in the prior 90 days, sedative load (cumulative effect of medications with sedative properties), and chronic opioid use in the prior year [22]. Health service utilisation was ascertained from the Medicare Benefits Schedule data 1 year prior to entry to long‐term home care. This included primary care (e.g., General Practitioner/medical practitioner attendances [regular, after hours and urgent after hours], optometrist attendances, specialist or consultant physician attendances [i.e., geriatrician], General Practitioner management plan, 75+ health assessments [only for individuals aged 75 years or older] and comprehensive medicines review).

Details of carer payments, such as carer payment (means‐tested, primary income support payment for individuals providing constant care for someone) and/or carer allowance (supplementary payments for carers providing care for someone who needs ongoing daily support) from the Australian Government [23], received by long‐term home care recipients (for those who completed a NSAF, 2016–2019) acting as an informal carer themselves were obtained from the DOMINO dataset [16].

Geographic variation in informal care was examined using Statistical Area Level 3 (SA3) regions [24] in Australia. The SA3 level was determined using the postcode reported on individuals' aged care eligibility assessments.

### 2.4 Statistical Analyses

The characteristics of those with and without access to informal carers were compared descriptively, with median and quartiles reported for continuous variables, and frequencies and percentages for categorical variables.

To investigate the change in the proportion of individuals reporting informal carer availability over time, the probability of having a carer was modelled using logistic regression with the date of receiving first long‐term home care service as an independent variable. This model was adjusted for age, sex and dementia to account for changes in care recipients' characteristics over time. The service date and age were modelled as continuous, linear independent variables and suitability of a linear relationship was assessed using restricted cubic splines. The effect of a 1‐year increase in receiving first long‐term home care service on the probability of having a carer was described using an odds ratio (OR) and 95% confidence interval (95% CI). The model was also used to estimate the probability of having a carer each year, standardised to the average population over the entire period, and presented in a plot with 95% CIs. Effect modification of time trends by age (modelled continuously and presented for ages 65, 70, 75, 80, 85 and 90), sex, dementia and culturally and linguistically or ethnically diverse status was examined, using logistic regression models with an interaction term between the date of service entry and each variable. Effect modification plots of predicted carer probability over time were presented.

Geographic variation by SA3 level, state, remoteness and socio‐economic disadvantage were compared for those with and without a carer. The proportion of long‐term home care recipients with a carer was calculated for each SA3 region with data for at least 100 participants (for robustness purposes) and displayed on a map of Australia.

## 3 Results

### 3.1 Cohort Description

Of the 240,282 care recipients eligible for inclusion (Figure S1), 6715 (3%) had missing or ‘not applicable’ carer status reported and were removed from our analysis. The characteristics of individuals with missing carer status were comparable to those with known carer status (Table S8).

Of the 233,567 care recipients with known carer status, 192,388 (82%) reported an informal carer. Care recipients with an informal carer were on average older (median age 83 vs. 81), more likely to be male (39% vs. 36%), speak a language other than English (13% vs. 6%) and have dementia (23% vs. 6%), a history of falls (17% vs. 12%) or Parkinson's disease (8% vs. 5%) compared to those without a carer (Table 1). In individuals with ACAP assessments between 2013 and 2016 (*n* = 123,394, 53%), most carers were daughters or daughters‐in‐law of the care recipient (37%), followed by wives/female partners (21%), husbands/male partners (16%) and sons/sons‐in‐law (16%). Fifty‐three per cent (*n* = 54,677) of carers lived with the care recipient, whereas 47% (*n* = 48,502) lived elsewhere (Table 2). In individuals that had NSAF assessments between 2016 and 2019 (*N* = 110,173, 47.2%), 15,509 (14.1%) care recipients reported that they were also acting as a carer for someone else. Of these, 43% were receiving carer payments at the time of assessment (Table S9).

**TABLE 1:** Characteristics of long‐term home care recipients by reported informal carer availability—*n* (%) except where indicated.

Characteristics	Has a carer *n* = 192,388 (82%)	Has no carer *n* = 41,179 (18%)	Overall *n* = 233,567 (100%)
Age, median (Q1, Q3)	83 (78, 88)	81 (75, 86)	83 (77, 87)
Sex			
Female	117,870 (61)	26,575 (65)	144,445 (62)
English as a preferred language^a^	165,740 (87)	38,520 (94)	204,260 (88)
Country of birth^a^			
Australia	120,463 (63)	28,431 (69)	148,894 (64)
Overseas	71,686 (37)	12,686 (31)	84,372 (36)
Home care package level approval^a^			
Level 1–2	94,645 (51)	28,972 (74)	123,617 (55)
Level 3–4	89,467 (49)	10,001 (26)	99,468 (45)
Living arrangements^a^			
Lives alone	70,779 (37)	28,362 (69)	99,141 (43)
Lives with family	117,861 (62)	11,737 (29)	129,598 (56)
Lives with others	2794 (2)	832 (2)	3626 (2)
Remoteness^a^			
Major cities	121,425 (63)	22,131 (54)	143,556 (62)
Inner regional	44,944 (24)	12,133 (30)	57,077 (25)
Outer regional	22,406 (12)	5946 (15)	28,352 (12)
Remote or very remote	2796 (2)	801 (2)	3597 (2)
SEIFA index of relative socio‐economic advantage and disadvantage^a^			
Quintile 1 (greater disadvantage)	34,051 (18)	9183 (23)	43,234 (19)
Quintile 2	36,367 (19)	8694 (21)	45,061 (19)
Quintile 3	36,999 (19)	8252 (20)	45,251 (20)
Quintile 4	35,471 (19)	6572 (16)	42,043 (18)
Quintile 5 (less disadvantage)	48,267 (25)	8193 (20)	56,460 (24)
Rx‐Risk‐V comorbidity category			
0–1	13,625 (7)	3232 (8)	16,857 (7)
2–3	34,734 (18)	7535 (18)	42,269 (18)
4–5	54,907 (29)	11,726 (29)	66,633 (29)
6–8	66,267 (34)	13,975 (34)	80,242 (34)
≥ 9	22,855 (12)	4711 (11)	27,566 (12)
Number of unique medications in previous 90 days			
0–4	52,068 (27)	11,339 (28)	63,407 (27)
5–10	98,904 (51)	21,141 (51)	120,045 (51)
≥ 11	41,416 (22)	8699 (21)	50,115 (22)
Antipsychotic use in previous 90 days	12,990 (6.8)	1851 (4.5)	14,841 (6)
High sedative load in previous year	67,038 (35)	15,511 (38)	82,549 (35)
Chronic opioid use in previous year	24,684 (13)	5685 (14)	30,369 (13)
Culturally and linguistically or ethnically diverse	47,659 (25)	7024 (17)	54,683 (24)
Health conditions^a^			
Dementia	44,537 (23)	2491 (6)	47,028 (20)
History of falls	31,923 (17)	5122 (12)	37,045 (16)
History of fractures	19,346 (10)	4239 (10)	23,585 (10)
History of pressure injury	8852 (5)	1959 (5)	10,811 (5)
Incontinence	27,275 (14)	4595 (11)	31,870 (14)
History of delirium	1895 (1)	232 (1)	2127 (1)
Epilepsy	11,237 (6)	2127 (5)	13,364 (6)
Parkinson's disease	15,599 (8)	2080 (5)	17,679 (8)
Stroke/cerebrovascular disease	30,003 (16)	4905 (12)	34,908 (15)
Diabetes	47,934 (25)	9878 (24)	57,812 (25)
Cancer	32,585 (17)	7045 (17)	39,630 (17)
Ischaemic heart disease	38,740 (20)	8003 (19)	46,743 (20)
Blindness	5385 (3)	998 (2)	6383 (3)
Deafness	26,366 (14)	4467 (11)	30,833 (13)
**Health services utilisation**			
General Practitioner/medical practitioner attendances in previous year			
0	7799 (4)	1724 (4)	9523 (4)
1–5	28,604 (15)	5011 (12)	33,615 (14)
6–15	89,491 (47)	17,863 (43)	107,354 (46)
≥ 16	66,494 (35)	16,581 (40)	83,075 (36)
General Practitioner/medical practitioner attendances after hours in previous year			
0	154,621 (80)	34,215 (83)	188,836 (81)
1	21,002 (11)	3871 (9)	24,873 (11)
2–4	12,203 (6)	2218 (5)	14,421 (6)
≥ 5	4562 (2)	875 (2)	5437 (2)
Urgent General Practitioner attendances after hours in previous year			
0	171,249 (89)	37,391 (91)	208,640 (89)
1	14,936 (8)	2667 (7)	17,603 (8)
2–4	5409 (3)	930 (2)	6339 (3)
≥ 5	794 (0)	191 (1)	985 (0)
Optometric services in previous year			
0	128,028 (67)	24,023 (58)	152,051 (65)
1	47,712 (25)	11,810 (29)	59,522 (26)
2–4	15,629 (8)	4956 (12)	20,585 (9)
≥ 5	1019 (1)	390 (1)	1409 (1)
Geriatric medicine attendances in previous year			
0	178,566 (93)	39,737 (97)	218,303 (94)
1	9049 (5)	964 (2)	10,013 (4)
≥ 2	4773 (3)	478 (1)	5251 (2)
General Practitioner management plan in previous year	106,970 (56)	24,461 (59)	131,431 (56)
75+ Health assessment in previous year^b^	55,016 (34)	11,780 (38)	66,796 (34)
Medicines review in previous year	9584 (5)	2160 (5)	11,744 (5)

*Note:* (Q1, Q3) denotes first and third quartiles.

Abbreviations: NSAF, National Screening and Assessment Form; SEIFA, Socio‐Economic Indexes for Areas.

^a^ Missing data: English as a preferred language (*n*=44); country of birth (*n*=19); home care package level approval (*n*=10,482); living arrangements (*n*=3360); remoteness (*n*=3150); SEIFA index of relative socio‐economic advantage and disadvantage (*n*=3158); culturally and linguistically or ethnically diverse (*n*=1345); health conditions (*n*=3185).

^b^ Health assessment in the previous year reported only for care recipients aged 75 and above.

**TABLE 2:** Characteristics of the relationship between long‐term home care recipients and their informal carer^a^.

Characteristics	Overall, *n* = 103,195
Carer co‐residency, *n* (%)^b^	
Co‐resident carer	54,677 (53)
Non‐resident carer	48,502 (47)
Carer relationship, *n* (%)^b^	
Wife/female partner	21,509 (21)
Husband/male partner	16,873 (16)
Daughter/daughter‐in‐law	38,615 (37)
Son/son‐in‐law	16,355 (16)
Other, female	7118 (7)
Other, male	2712 (3)
Respite care use by long‐term home care recipient or carer, *n* (%)^b^	
None	92,999 (90)
Residential respite care	3827 (4)
Non‐residential respite care	5157 (5)
Residential and non‐residential respite care	857 (1)

^a^ Only includes care recipients with an aged care eligibility assessment using an ACAP (2013–2016) assessment (*n* = 123,394 with known carer status) who reported having a carer (*n* = 103,195).

^b^ Missing data: carer co‐residency (*n* = 16); carer relationship (*n* = 13); respite care use (*n* = 355).

### 3.2 Time Trends

**FIGURE 1:**
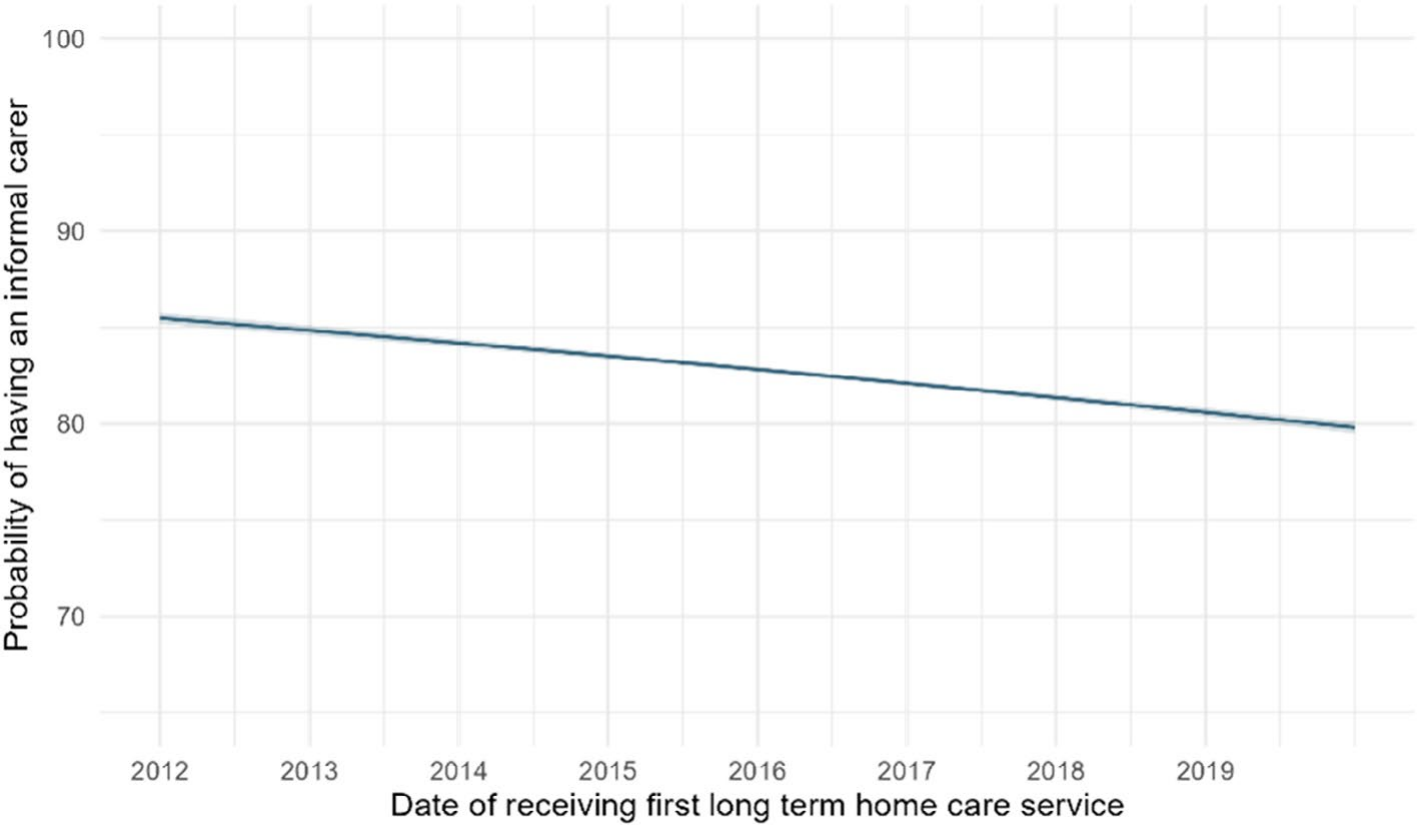
Predicted probability of having an informal carer by date of receiving first long‐term home care service, adjusted for age, sex and dementia status. Predicted probabilities obtained from logistic regression model adjusted for age, sex and dementia using standardisation. 95% confidence intervals are indicated by the shaded region.

**FIGURE 2:**
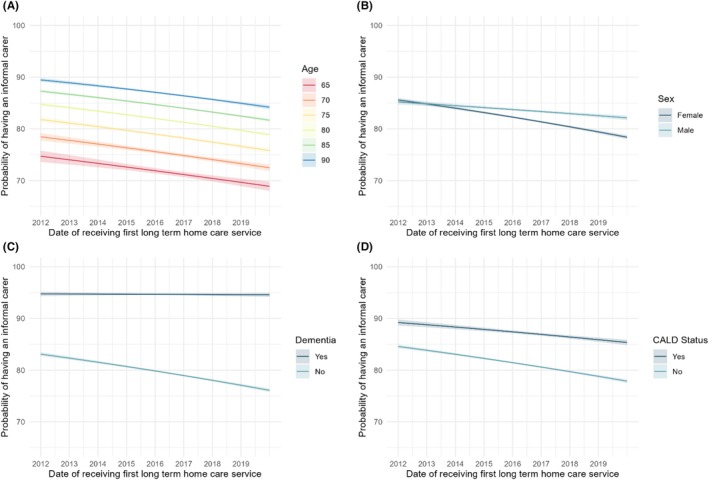
Predicted probability of having an informal carer by date of receiving first long‐term home care service, by (A) age; (B) sex; (C) dementia; (D) culturally and linguistically or ethnically diverse (CALD) status. Predicted probabilities were obtained using standardisation, from logistic regression model with interaction terms between date of receiving first long‐term home care service and each of age, sex, dementia and CALD status. 95% confidence intervals are indicated by the shaded regions.

The proportion of long‐term home care recipients with an informal carer decreased from 86% in 2012 to 78% in 2019 (adjusted OR: 0.95, 95% CI 0.95–0.95) (Figure 1). There was evidence that the time trend in reported informal carer availability varied depending on age, sex, dementia, and culturally and linguistically or ethnically diverse status of care recipients, but not remoteness (Table S10). The decrease in carers over time was slightly more pronounced for older care recipients, in females (OR: 0.96, 95% CI 0.95–0.97) compared with males (OR: 0.99, 95% CI 0.98–1.00), in care recipients without dementia (OR: 0.95, 95% CI 0.94–0.95) compared to those living with dementia (OR: 1.00, 95% CI 0.98–1.02), and in those who were not culturally and linguistically or ethnically diverse (OR: 0.94, 95% CI 0.94–0.95) compared with those who were (OR: 0.96, 95% CI 0.95–0.97) (Figure 2 and Table S10).

### 3.3 Geographic Variation

Individuals in major cities were more likely to have reported informal carer availability than those in regional/remote areas (85% in major cities vs. 78% in remote areas). Those living in more disadvantaged areas according to the SEIFA Index had lower levels of informal carer availability (79% in most disadvantaged quintile vs. 86% in most advantaged quintile). State and territory differences are displayed in Table 1.

**FIGURE 3:**
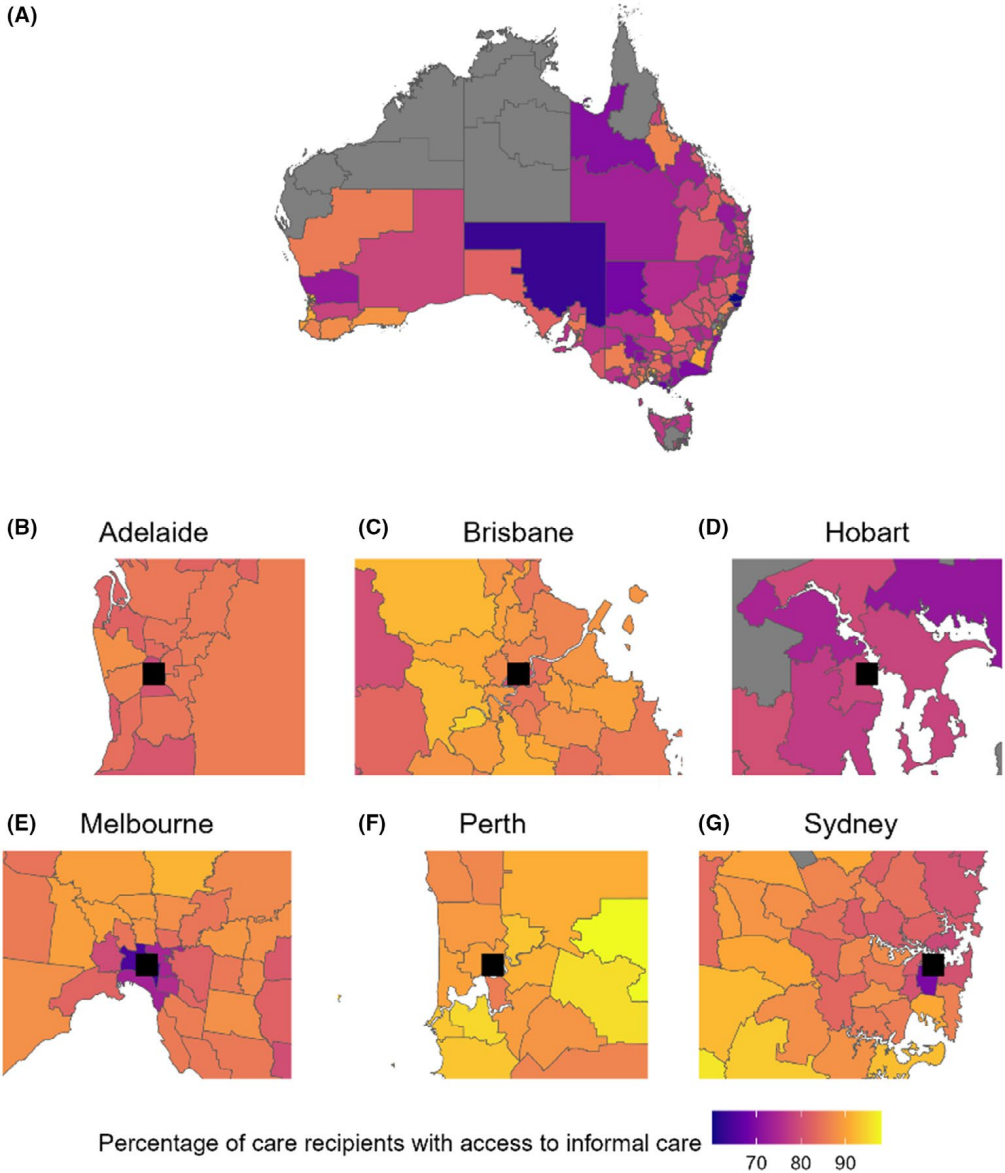
Variation in percentage of long‐term home care recipients with an informal carer in Australia by Statistical Area Level 3. The map shows variation for the whole of Australia (A) and in magnified regions to show capital cities of (B) Adelaide, (C) Brisbane, (D) Hobart, (E) Melbourne, (F) Perth and (G) Sydney. The black square represents the central business district of each city. Grey areas indicate Statistical Area Level 3 regions with data for < 100 care recipients recorded. The shaded colour of region represents the percentage of long‐term home care recipients with an informal carer, ranging from dark purple (60%) to bright yellow (98%).

The proportion of long‐term home care recipients with an informal carer varied considerably by SA3 regions, ranging from 60% to 98%. Visualisations of informal care prevalence show metropolitan areas with lower levels in city centres. The percentages of reported informal carers in Melbourne City (65%) and Inner‐City Sydney (69%) were the 4th and 7th lowest in the country, respectively (Figure 3).

## 4 Discussion

This is the first national population‐based study to characterise informal carer availability and support in older people initiating long‐term home care support in Australia. We found that a high proportion of long‐term home care recipients reported an informal carer (82%) between 2012 and 2019. However, the proportion of care recipients with an informal carer decreased from 86% in 2012 to 78% in 2019 after adjustment for cohort changes in age, sex, and dementia. The decrease in informal carers reported over time was slightly higher in females and individuals without dementia. In individuals with the most recent aged care eligibility assessments (between 2016 and 2019), 14% were also caring for someone else. We also found considerable national variation in informal carer availability, particularly with lower levels of carers in socio‐economically disadvantaged areas, remote regions and metropolitan city centres.

Our study found a decline in reported informal carer availability for older people in long‐term home care during the study period, highlighting the need for policy and care planning to address these changes. This finding was inconsistent with the Survey of Disability, Ageing and Carers report, which found a slight increase in the proportion of carers from 10% in 2018 to 12% of the population in 2022. This difference may be because the Survey of Disability, Ageing and Carers only included people who were living in households and providing unpaid care, with prevalence estimates over time not adjusted for age, sex or dementia status. In our study, only half of informal carers lived with the care recipient. However, previous projections in 2020 suggest a potential decline in the availability of family members to provide unpaid care in the future, likely due to changing family structures and increased geographical mobility [2]. It is concerning that the proportion of carers significantly decreased over time, particularly among older people, females and those without dementia. The difference in life expectancy between males and females may have contributed to the more pronounced decline in informal carer availability over time for female care recipients [25]. It is also possible that more people are opting to live at home alone instead of going to residential care, particularly as access to residential care has declined in recent years [26].

Our study identified substantial geographic disparities in reported informal carer availability for older people in long‐term home care across Australia. We found lower levels of reported informal carer availability in regional and remote areas compared with major cities (78% vs. 85%), a difference likely due to higher population densities and proximity to family members in major cities [21, 27, 28]. However, we identified disparities within metropolitan areas, in particular lower levels of reported informal carer availability in city centres highlighting unique challenges in urban environments, which are lower than the proportion in regional and remote areas [15, 25, 29]. This may be driven by a higher proportion of younger working‐age populations with limited time available for caregiving roles, limited housing space or less access to family or social networks [15]. Urban lifestyles may also contribute to greater reliance on formal care services, with some older people moving to city centres to access health care services and subsequently requiring long‐term home care [29]. Additionally, a recent national report has shown that older people with an informal carer had longer wait times to receive their approved long‐term home care service than people without an informal carer, highlighting the importance of informal carers while waiting for access to formal services [30]. Long‐term home care recipients living in disadvantaged areas (79% in the most disadvantaged quintile vs. 86% in the most advantaged) had lower levels of reported informal carer availability, likely because economic constraints in these areas may reduce the capacity for family members to provide unpaid care, consistent with previous literature [12]. Our study supports previous reports that economic and social inequalities influence informal care patterns [31, 32].

Our findings highlight that long‐term home care recipients with informal carers are more likely to have greater care needs, be older, have dementia, have a history of falls or Parkinson's disease, and be from culturally and linguistically diverse backgrounds. These findings underscore the critical role of informal carers in supporting older Australians with complex needs to remain living at home and emphasise the importance of policies and services that recognise and strengthen informal care networks. Given that most older people prefer to remain living independently at home as long as possible [33], ensuring adequate support and the availability of informal carers remains critical to support safe and successful ageing in place [25]. Additionally, our finding of substantial geographic disparities in reported informal carer availability for older people in long‐term home care across Australia suggests a need for targeted policy interventions and support systems to address socio‐economically disadvantaged areas, remote regions and metropolitan city centres, such as through financial incentives, increased respite care services or community‐led programmes [8].

Our study has some limitations. First, care recipients with missing informal carer availability (3%) were removed from the analysis. However, characteristics of care recipients with missing carer status were found to be comparable to those with known carer status. Second, our study relied on responses provided by care recipients during their aged care eligibility assessment by clinically trained independent assessors. Evidence suggests that not all people who provide informal care always identify themselves as carers due to cultural reasons or real or perceived stigma [21]. Third, there was variation in the definition of carer status captured in the two data collection tools used for aged care eligibility assessments that were included in this study. However, this change only had a minor impact on the overall trend of reported informal carer availability (Figure S3). Fourth, there was a median 4‐month delay between completing the aged care eligibility assessment and receiving long‐term home care and it is possible that informal carer status may have changed (Table S1). A further limitation is the lack of information in our datasets regarding the type, quality and extent of care provided by informal carers, including the number of caregiving hours, level of carer stress and the nature of support provided. These factors could be important when estimating the overall reliance on informal care.

## 5 Conclusions

Our study highlights the critical role of informal carers in supporting long‐term home care recipients in Australia, despite a significant decline in their availability between 2012 and 2019. We found that 14% of care recipients were also providing informal care to others, balancing their own care needs while simultaneously providing informal care to others. Our study also identified substantial national geographical variations in reported informal carer availability, with lower rates in socio‐economically disadvantaged areas, remote regions and metropolitan city centres. Lower informal carer availability likely translates in greater formal care needs by individuals in these areas and aged care sector demands.

## Funding

This work was supported with the financial and other support of the Health Translation SA (HTSA) Medical Research Future Fund Catalyst Grant Scheme, led by HTSA, supported by the Hospital Research Foundation Group. G.E.C. (GNT2026400), J.K.S. (GNT2016277) and M.C.I. (GNT119378) are supported by the National Health and Medical Research Council.

## Ethics Statement

Approval was obtained from the University of South Australia Human Research Ethics Committee (Ref: 200489) and the Australian Institute of Health and Welfare Ethics Committee (Ref: EO2022/4/1376).

## Conflicts of Interest

J.K.S. is a Non‐Executive Director of Southern Cross Care SA, NT & VIC (aged care provider organisation). All other authors declare no conflicts of interest.

## Supporting information


**Table S1:** Time between ACAP/NSAF assessment and receiving first long‐term home care.
**Table S2:** Percentage of care recipients with an informal carer by aged care eligibility assessment type (entire time period).
**Table S3:** Percentage of care recipients with an informal carer by aged care eligibility assessment type (assessments in 2015–2016 only).
**Table S4:** Definition of variables used to characterise long‐term home care recipients.
**Table S5:** Classification of long‐term home care recipient health conditions using Rx‐Risk and health condition codes reported at the time of aged care eligibility assessments.
**Table S6:** Number of care recipients classified as having a health condition using ACAP/NSAF and Rx‐Risk‐V definitions.
**Table S7:** Medicare Benefits Schedule item numbers used to classify health service utilisation characteristics.
**Table S8:** Characteristics of care recipients with missing carer status versus those with non‐missing carer status.
**Table S9:** Long‐term home care recipients receiving carer payments by whether they reported being a carer.
**Table S10:** Effect of 1‐year increase in date of receiving first long‐term home care service on probability of having an informal carer by age, sex and dementia status.
**Figure S1:** Study flow chart.
**Figure S2:** Proportion of ACAP and NSAF assessments by year of assessment.
**Figure S3:** Proportion of care recipients with an informal carer by year of assessment.
**Methods S1** Ascertainment of informal care availability from aged care eligibility assessments.

## Data Availability

The data that support the findings of this study were made available to the researchers under ethical, governance and confidentiality agreements that do not allow public sharing.
