# Supplementary material for: Characterising Informal Care in Older Individuals Receiving Long‐Term Home Care Support: A National Epidemiological Study
Source: Australas J Ageing. 2026 Jan 22;45(1):e70128. doi: 10.1111/ajag.70128 (PMC12824823; doi:10.1111/ajag.70128)
Supplement: Supplementary file 1 — Table S1: Time between ACAP/NSAF assessment and receiving first long‐term home care. Table S2: Percentage of care recipients with an informal carer by aged care eligibility assessment type (entire time period). Table S3: Percentage of care recipients with an informal carer by aged care eligibility assessment type (assessments in 2015–2016 only). Table S4: Definition of variables used to characterise long‐term home care recipients. Table S5: Classification of long‐term home care recipient health conditions using Rx‐Risk and health condition codes reported at the time of aged care eligibility assessments. Table S6: Number of care recipients classified as having a health condition using ACAP/NSAF and Rx‐Risk‐V definitions. Table S7: Medicare Benefits Schedule item numbers used to classify health service utilisation characteristics. Table S8: Characteristics of care recipients with missing carer status versus those with non‐missing carer status. Table S9: Long‐term home care recipients receiving carer payments by whether they reported being a carer. Table S10: Effect of 1‐year increase in date of receiving first long‐term home care service on probability of having an informal carer by age, sex and dementia status. Figure S1: Study flow chart. Figure S2: Proportion of ACAP and NSAF assessments by year of assessment. Figure S3: Proportion of care recipients with an informal carer by year of assessment. Methods S1 Ascertainment of informal care availability from aged care eligibility assessments. [file AJAG-45-0-s001.docx]

**Supplementary Data**

**Contents list**

[**Supplementary Methods 1.** Ascertainment of informal care availability from aged care eligibility assessments 3](#_Toc192087778)

[**Supplementary Table 1.** Time between ACAP/NSAF assessment and receiving first long-term home care………………………………………………………………………4](#_Toc192087782)

[**Supplementary Table 2.** Percentage of care recipients with an informal carer by aged care eligibility assessment type (entire time period). 6](#_Toc192087783)

[**Supplementary Table 3.** Percentage of care recipients with an informal carer by aged care eligibility assessment type (assessments in 2015 - 2016 only). 6](#_Toc192087784)

[**Supplementary Table 4.** Definition of variables used to characterise long-term home care recipients. 8](#_Toc192087785)

[**Supplementary Table 5.** Classification of long-term home care recipient health conditions using Rx-Risk and health condition codes reported at the time of aged care eligibility assessments. 10](#_Toc192087786)

[**Supplementary Table 6.** Number of care recipients classified as having a health condition using ACAP/NSAF and Rx-Risk-V definitions. 11](#_Toc192087787)

[**Supplementary Table 7.** Medicare Benefits Schedule item numbers used to classify health service utilisation characteristics. 11](#_Toc192087788)

[**Supplementary Table 8.** Characteristics of care recipients with missing carer status versus those with non-missing carer status. 13](#_Toc192087789)

[**Supplementary Table 9**. Long-term home care recipients receiving carer payments by whether they reported being a carer. 17](#_Toc192087790)

[**Supplementary Table 10.** Effect of one-year increase in date of receiving first long-term home care service on probability of having an informal carer by age, sex, and dementia status. 18](#_Toc192087791)

[**Supplementary Figure 1.** Study flow chart………………………………………………..2](#_Toc192087792)

[**Supplementary Figure 2.** Proportion of ACAP and NSAF assessments by year of assessment. 7](#_Toc192087793)

[**Supplementary Figure 3.** Proportion of care recipients with an informal carer by year of assessment. 7](#_Toc192087794)

**
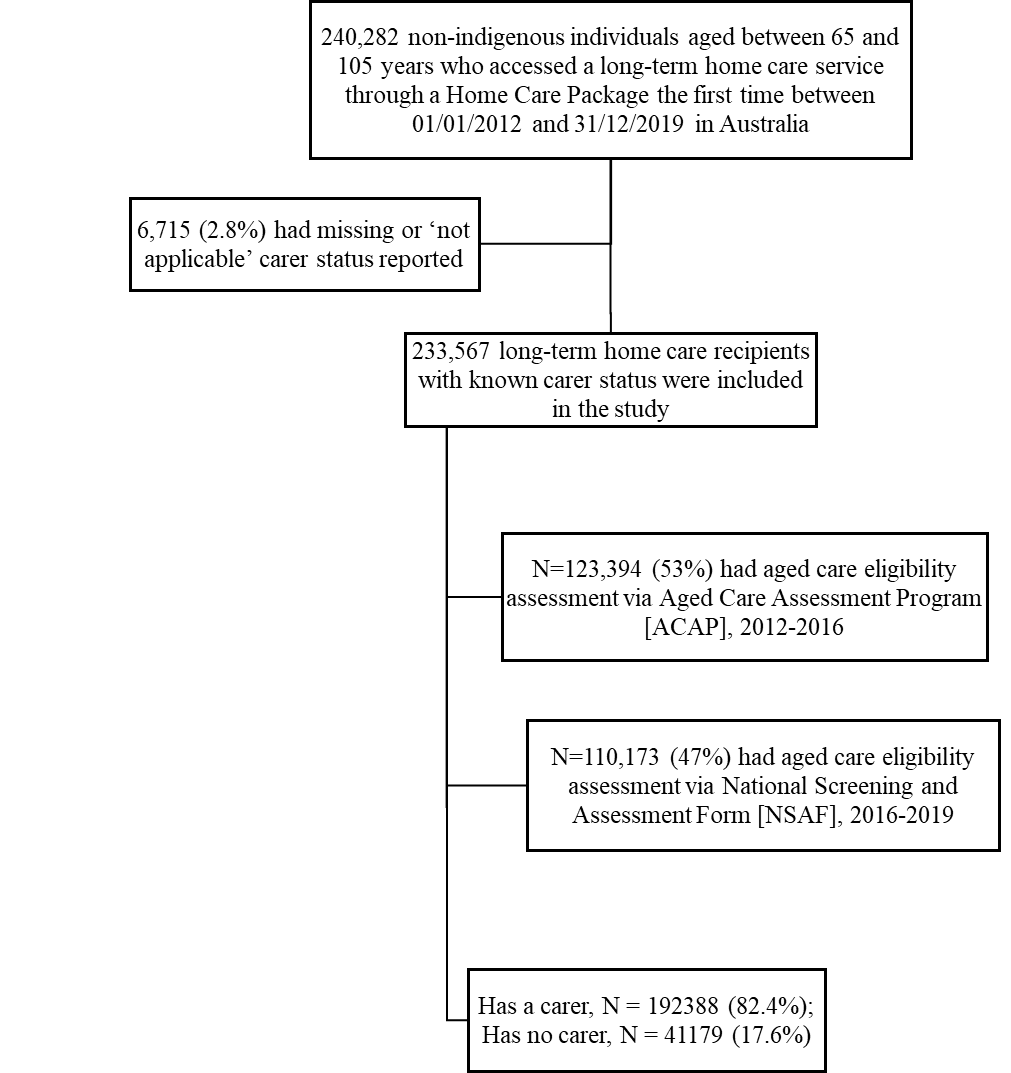
**

**Supplementary Figure** **1:** Study flow chart

**Supplementary Methods 1**: Ascertainment of informal care availability from aged care eligibility assessments

Care recipient information, including informal care availability, was ascertained using information provided in the individual’s most recent aged care eligibility assessment (i.e. Aged Care Assessment Program [ACAP] and National Screening and Assessment Form [NSAF]) prior to the date of receiving their first long-term home care. In 2016, the assessment form for accessing aged care switched from the ACAP to NSAF. If both an ACAP and NSAF assessment were completed on the same day, then the NSAF assessment was used. Where more than one assessment of the same type was completed on the same day, the information from both assessments was combined as follows: (1) For each question, if one assessment had missing information and the other had complete information, then the answer from the assessment with complete information was used; (2) If the same question was answered in both assessments, but the answers given were different, then the answer was considered unknown and set to blank,; and (3) If the same question was answered in both assessments, but the answers given were identical, then no action was required.

**Time lag between assessment and receiving first long-term home care**

There is a wait time for receiving long term home care after completing an aged care eligibility assessment. If wait times are long, this may raise concerns that care recipients’ information is no longer current at the time of receiving their first home care package (the point of entry into this study). To address this concern, we examined the time between each care recipient’s most recent assessment and their first instance of long-term home care. We found that the median time was four months, with 75% of care recipients receiving their first long-term home care within one year of most recent assessment, and 92% within two years. Times appeared similar for those with and without a carer (**Supplementary Table 1**).

(Note that the purpose of this analysis was to review how current the information was at the time of receiving a home care package and not to examine wait times. As we have used the time from the *most recent* assessment this may not reflect the actual wait time).

**Supplementary Table 1:** Time between ACAP/NSAF assessment and receiving first long-term home care

| **Characteristics** | **Has a carer**  **n = 192,388 (82%)** | **Has no carer**  **n = 41,179 (18%)** | **Overall,**  **n = 233,567 (100%)** |
| --- | --- | --- | --- |
| Time between assessment and receiving first long-term home care (months), median (Q1, Q3) | 4.0 (1.1,12.1) | 4.6 (1.1, 11.7) | 4.1 (1.1, 12.0) |
| Time between assessment and receiving first long-term home care, n (%) |  |  |  |
| 0-1 years | 143,894 (75) | 31,180 (76) | 177,462 (75) |
| 1-2 years | 33,251 (17) | 6,719 (16) | 41,030 (17) |
| 2-3 years | 7,885 (4) | 1,662 (4) | 9,635 (4) |
| 3-4 years | 4,148 (2) | 921 (2) | 5,101 (2) |
| 4-5 years | 1,927 (1) | 428 (1) | 2,358 (1) |
| 5+ years | 1,283 (1) | 269 (1) | 1,568 (1) |

Note: (Q1, Q3) denotes first and third quartiles.

**Classification of informal care availability from ACAP and NSAF assessments**

While both assessments captured information about informal care, the question wording changed in NSAF as follows:

**ACAP:** A carer defined as someone, such as a family member, friend or neighbour, excluding paid or volunteer carers organised by formal services, has been identified as providing regular and sustained care and assistance to the person without payment other than a pension or benefit.

**NSAF:** Whether the client is receiving assistance from a carer, family member(s), friend(s) and/or neighbour(s) not associated with a service provider or paid service. To avoid doubt, an individual is not a carer merely because he or she: (1) Is the spouse, de facto partner, parent, child or other relative of an individual, or (2) Is the guardian of an individual; or lives with an individual who requires care.

To examine the impact of the slight change in definition on the prevalence of informal care, particularly the time trends, we compared the proportion of care recipients with and without informal care by the type of assessment tool used (**Supplementary Table 2**). When restricting the data to assessments that took place in 2015/2016 (when both assessments were in use), recipients who completed an NSAF assessment showed a slightly higher proportion of informal care compared to the ACAP (84% in NSAF versus 81% in ACAP) (**Supplementary Table 3**). However, this change only had a minor impact on the overall downward trend of informal care seen in **Supplementary Figure 3.**

**Supplementary Table 2:** Percentage of care recipients with an informal carer by aged care eligibility assessment type (entire time period)

| **Carer availability** | **ACAP**  **(n = 123,650)** | **NSAF**  **(n = 113,504)** | **Overall**  **(n = 240,282)** |
| --- | --- | --- | --- |
| Has a carer | 103,195 (84) | 89,193 (81) | 192,388 (82) |
| Has no carer | 20,199 (16) | 20,980 (19) | 41,179 (18) |

**Supplementary Table 3:** Percentage of care recipients with an informal carer by aged care eligibility assessment type (assessments in 2015 - 2016 only).

| **Carer availability** | **ACAP**  **(n = 37,921)** | **NSAF**  **(n = 30,380)** | **Overall**  **(n = 68,301)** |
| --- | --- | --- | --- |
| Has a carer | 30,779 (81) | 24,773 (84) | 55,552 (83) |
| Has no carer | 7,099 (19) | 4,630 (16) | 11,729 (17) |


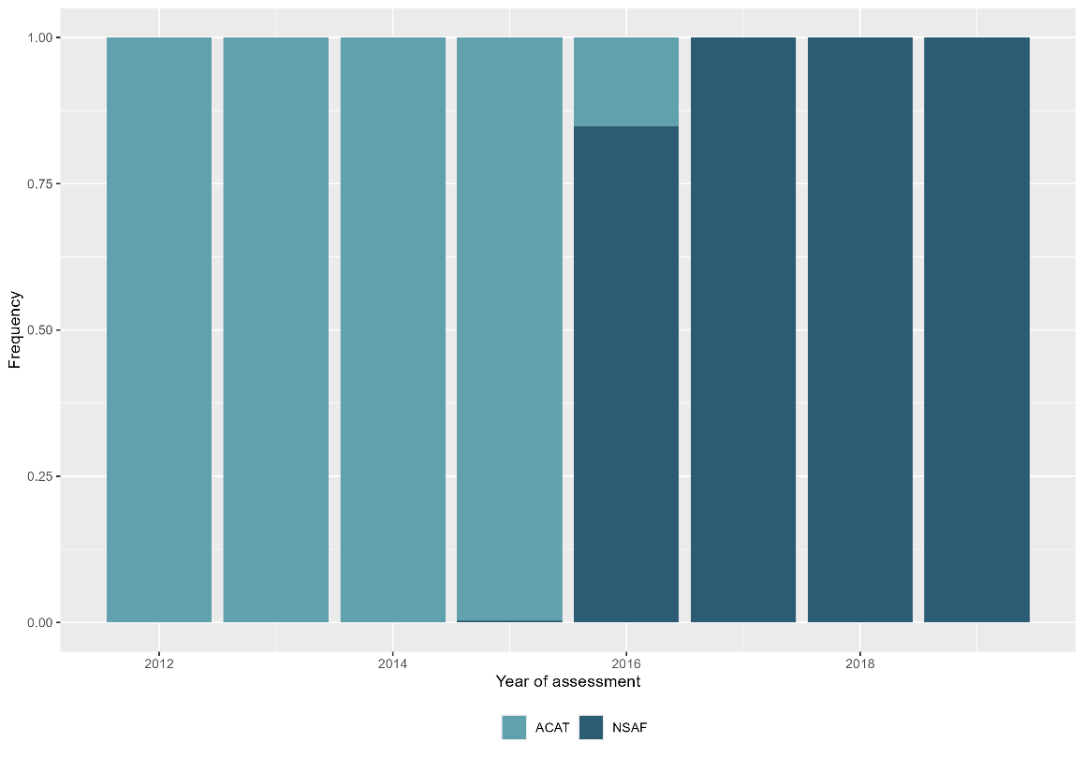


**Supplementary Figure 2:** Proportion of ACAP and NSAF assessments by year of assessment


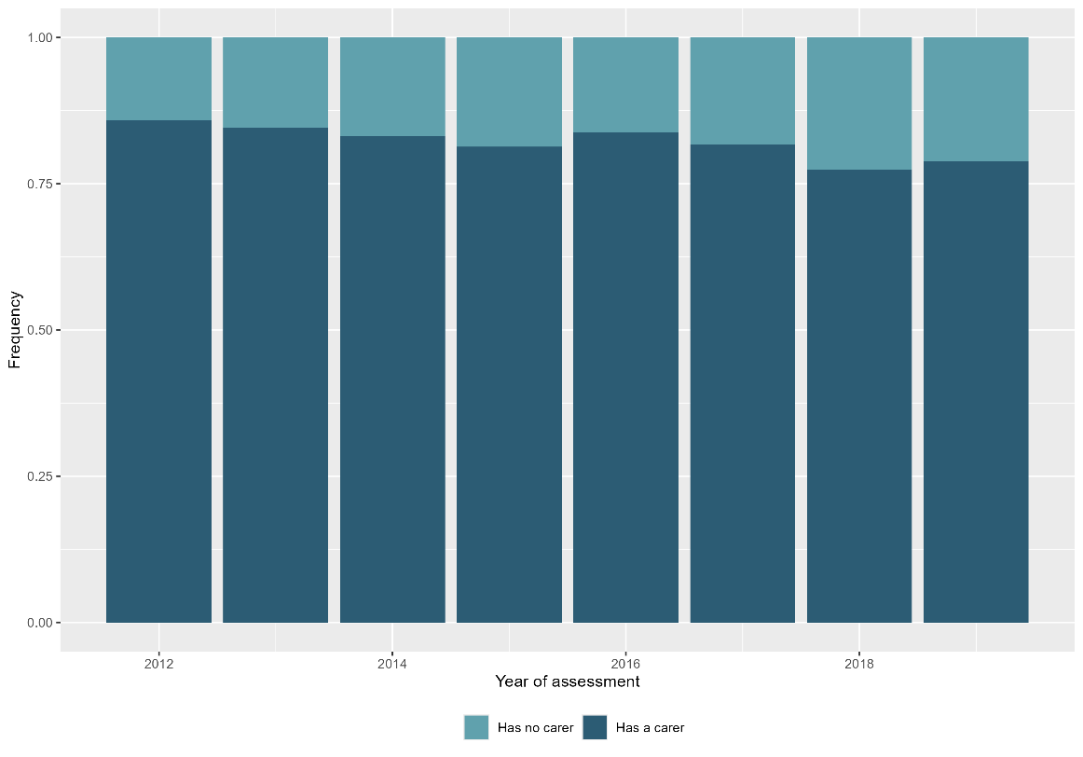


**Supplementary Figure 3:** Proportion of care recipients with an informal carer by year of assessment

**Supplementary Table 4:** Definition of variables used to characterise long-term home care recipients

| **Characteristics** | **Definition** | |
| --- | --- | --- |
| Culturally or linguistically diverse status | Culturally or linguistically diverse status was determined using country of birth and preferred language.^1^ Care recipients were considered to be from a Culturally or linguistically diverse background if they either:   - Reported a preferred language other than English - Were born in a country other than Australia, United Kingdom (England, Scotland, Wales, Northern Ireland), Republic of Ireland, New Zealand, Canada, United States of America or South Africa.^2^ | |
| Number of unique medicines | Using the PBS data, the number of unique medicines with prescriptions filled in the 90 days prior to the date first long-term home care service was received, based on ATC codes at the 5th level (7 digits). Records with missing ATC code, or codes of ‘Z’ or beginning with ‘V’ were not included in the count. | |
| Antipsychotic use | History of dispensing of antipsychotic medications (i.e., ATC codes beginning with “N05A” [excluding “N05AB04”, “N05AN01”, “N05AN”]) in the 90 days prior to the date first long-term home care service was received. | |
| Chronic opioid use | Chronic opioid use was defined as receiving any number of opioid medications for at least 90 days continuously, or for 120 non-consecutive days in the year prior to receiving first long-term home care. No gap days between one opioid medication dispensing and another were allowed when determining consecutive use of opioids.^3^ |  |
| High sedative load | Care recipients were considered to have a high sedative load if their calculated sedative load was high in any of the quarters (~91-day periods) in the year preceding receipt of their first long-term home care, consistent with the ROSA OMS specifications.^3^ For each quarter, sedative load was calculated by summing the sedative rating of each different medication dispensed within the ~91-day time period prior to receiving their first long-term home care.^3^ Each drug was only counted once towards sedative load within the 90-day period regardless of the number of scripts or quantity dispensed. |  |
| Rx-Risk-V comorbidity category | A care recipient was considered to have a health condition according to the Rx-Risk definition if they were dispensed at least one medicine indicative of that condition in the six months prior to the date of receiving their first long-term home care. The number of conditions each care recipient had was summed and reported as the Rx-Risk-V comorbidity category.^4^ |  |
| Health conditions | Care recipients were considered to have a health condition if either of the following two conditions were met: (1) a health condition code in their ACAP/NSAF corresponding to the health condition was reported; (2) They were prescribed a medication in the previous six months indicative of that health condition according to Rx-Risk-V classification (**Supplementary Table 5**). |  |
| Health service utilisation in previous year | Care recipients were considered to have utilised a service if they had an MBS item number corresponding to the codes in **Supplementary Table 7** with a date in the year prior to their first long-term home care. A care recipient was assumed to have utilised a service zero times if there were no MBS records of that item number recorded in the previous year. |  |

MBS: Medicare Benefits Schedule; PBS: Pharmaceutical Benefits Scheme; ACAP: Aged Care Assessment Program; NSAF: National Screening and Assessment Form. ATC: The World Health Organisation Anatomic-Therapeutic Classification codes.

^1^Australian Bureau of Statistics. 3415.0-Migrant Data Matrices. Available online: [*https://www.abs.gov.au/AUSSTATS/abs@.nsf/Lookup/3415.0Glossary12013?OpenDocument*](https://www.abs.gov.au/AUSSTATS/abs@.nsf/Lookup/3415.0Glossary12013?OpenDocument) (Viewed December 2024).

^2^Australian Bureau of Statistics. Standard Australian Classification of Countries (SACC). Available online: <https://www.abs.gov.au/statistics/classifications/standard-australian-classification-countries-sacc/latest-release#data-downloads> (Viewed December 2024).

^3^Registry of Senior Australians. ROSA Outcome Monitoring System Technical Specification. South Australian Health and Medical Research Institute. Available online: <https://sahmri.org.au/research/programs/registry-centre/groups/rosa/rosa-oms> (Viewed December 2024).

^4^Pratt NL, Kerr M, Barratt JD, et al. The validity of the Rx-Risk Comorbidity Index using medicines mapped to the Anatomical Therapeutic Chemical (ATC) Classification System. BMJ Open. 2018;8(4):e021122.

**Supplementary Table 5:** Classification of long-term home care recipient health conditions using Rx-Risk and health condition codes reported at the time of aged care eligibility assessments

| **Health conditions** | **ACAP/NSAF health condition codes** | **Rx-Risk-V*** |
| --- | --- | --- |
| Diabetes | 0403, 0402, 0404 | 13 |
| Dementia | 0500, 0501, 0502, 0503, 0504, 0510, 0511, 0512, 0513, 0514, 0515, 0516, 0520, 0521, 0522, 0523, 0526, 0530, 0531, 0532, 0584 | 11 |
| Falls | 1715 | NA |
| Fracture | 1606, 1607, 1608, 1609, 1610, 1611, 1612 | NA |
| Incontinence | 1403, 1717, 1708 | 29 |
| Delirium | 0540, 0541, 0542, 0543, 0544 |  |
| Epilepsy | 0608 | 14 |
| Parkinson’s | 0604 | 38 |
| Pressure injuries | 1201, 1299 | NA |
| Cancer | 0201, 0202, 0203, 0204, 0205, 0206, 0207, 0208, 0209, 0210, 0211, 0212, 0213, 0214, 0215, 0216, 0217, 0299 | 32 |
| Blindness | 0703 | NA |
| Deafness | 0802 | NA |
| Stroke/cerebrovascular disease | 0910, 0911, 0912, 0913, 0914, 0915, 0916, 0605 | NA |
| Ischaemic heart disease | 0903, 0904, 0905 | 27 |

ACAP: Aged Care Assessment Program; NSAF: National Screening and Assessment Form; NA: Not Available. *Note an Rx-Risk-V code of NA indicates that this health condition was defined using ACAP/NSAF health condition codes only. For health conditions defined using a combination of Rx-Risk and aged care assessment health condition codes, the number classified according to each system are detailed in **Supplementary Table 6**

**Supplementary Table 6:** Number of care recipients classified as having a health condition using ACAP/NSAF and Rx-Risk-V definitions, n (%)

| **Health Condition** | **ACAP/NSAF health condition code only** | **Rx-risk only** | **Both** | **Neither** |
| --- | --- | --- | --- | --- |
| Diabetes | 12,128 (5) | 8,448 (4) | 37,236 (16) | 175,672 (75) |
| Dementia | 22,558 (10) | 5,958 (3) | 18,512 (8) | 186,456 (80) |
| Incontinence | 22,342 (10) | 7,114 (3) | 2,414 (1) | 201,614 (86) |
| Epilepsy | 386 (0) | 10,161 (4) | 2,817 (1) | 220,120 (94) |
| Parkinson’s | 1,362 (1) | 6,256 (3) | 10,061 (4) | 215,805 (92) |
| Cancer | 31,383 (13) | 5,394 (2) | 2,853 (1) | 193,854 (83) |
| Ischaemic heart disease | 21,684 (9) | 14,250 (6) | 10,809 (5) | 186,741 (80) |

ACAP: Aged Care Assessment Program; NSAF: National Screening and Assessment Form.

**Supplementary Table 7:** Medicare Benefits Schedule item numbers used to classify health service utilisation characteristics

| Health care service | MBS group | Description of MBS Group | Included MBS Items |
| --- | --- | --- | --- |
| GP/Medical practitioner attendances | A01 | GP attendance | 3, 4, 23, 24, 36, 37, 44, 47 |
|  | A02 | Non-referred attendance to medical practitioner | 52, 53, 54 ,57, 58, 59, 60, 65 |
|  | A35 | Medical services at residential aged care facilities | 20, 35, 43, 51, 92, 93, 95, 96, 183, 188, 202, 212, 90020, 90035, 90043, 90051, 90092, 90093, 90095, 90096, 90183, 90188, 90202, 90212 |
| GP/Medical practitioner after-hours attendances | A22 | GP after-hours attendance | 5000, 5003, 5010, 5020, 5023, 5028, 5040, 5043, 5049, 5060, 5063, 5067 |
|  | A23 | Non-referred after-hours attendance with medical practitioners | 5200, 5203, 5207, 5208, 5220, 5223, 5227, 5228, 5260, 5263, 5265, 5267 |
| Urgent GP attendance after-hours | A11 | Urgent GP after-hours attendance | 585, 588, 591, 594, 597, 598, 599, 600 |
| Health assessments | A14 | GP/Medical practitioner health assessments | 224, 225, 226, 227, 701, 703, 705, 707 |
| Management plans | A15 | GP management plan attendances/team care arrangements and multidisciplinary care plans | 229, 230, 231, 232, 233, 235, 236, 237, 238, 239, 240, 243, 244, 721, 723, 729, 731, 732, 735, 739, 743, 747, 750, 758, 871, 872 |
| Geriatric medicine | A28 | Geriatric medicine attendances | 141, 143, 145, 147, 149 |
| Optometric services | A10 | Optometric services | 10905, 10907, 10911, 10912, 10913, 10915, 10916, 10918, 10922, 10923, 10924, 10925, 10926, 10927, 10928, 10929, 10930, 10931, 10932, 10933, 10940, 10941, 10942, 10943, 10944, 10945, 10946, 10947, 10948 |

MBS: Medicare Benefits Schedule; GP: general practitioner.

**Missing Carer Status**

A small proportion of the study cohort had missing (blank, ‘Not applicable’, or ‘Not stated’) carer status. These records were excluded from the analysis. To check whether the care recipients included in the study were representative of those excluded, the characteristics of care recipients with missing and non-missing carer status were compared descriptively.

**Supplementary Table 8:** Characteristics of care recipients with missing carer status versus those with non-missing carer status.

| **Characteristics** | **Missing (n = 6,715)** | **Not missing (n = 233,567)** |
| --- | --- | --- |
| **Age, median (Q1, Q3)** | 81.0 (73.0-87.0) | 83.0 (77.0-87.0) |
| **Sex, n (%)** |  |  |
| Female | 4,110 (61) | 144,445 (62) |
| **Preferred language, n (%)** |  |  |
| English | 5,835 (88) | 204,260 (88) |
| Others | 836 (13) | 27,698 (12) |
| **Country of birth, n (%)** |  |  |
| Australia | 4,290 (64) | 148,894 (64) |
| Overseas | 2,406 (36) | 84,372 (36) |
| **Living arrangements, n (%)** |  |  |
| Lives alone | 1,463 (44) | 99,141 (43) |
| Lives with family | 1,808 (54) | 129,598 (56) |
| Lives with others | 84 (3) | 3,626 (2) |
| **State, n (%)** |  |  |
| Australian Capital Territory | 44 (1) | 3,702 (2) |
| New South Wales | 1,290 (36) | 81,258 (35) |
| Northern Territory | 13 (0) | 796 (0) |
| Queensland | 769 (22) | 46,591 (20) |
| South Australia | 297 (8) | 18,832 (8) |
| Tasmania | 120 (3) | 5,682 (2) |
| Victoria | 707 (20) | 51,948 (22) |
| Western Australia | 330 (9) | 24,191 (10) |
| **ARIA remoteness, n (%)** |  |  |
| Major cities | 2,236 (63) | 143,556 (62) |
| Inner regional | 850 (24) | 57,077 (25) |
| Outer regional | 424 (12) | 28,352 (12) |
| Remote or very remote | 55 (2) | 3,597 (2) |
| **Index of Relative Socio-economic Advantage and Disadvantage quintile, n (%)** |  |  |
| Quintile 1 (greater disadvantage) | 620 (17) | 43,234 (19) |
| Quintile 2 | 674 (19) | 45,061 (19) |
| Quintile 3 | 717 (20) | 45,251 (20) |
| Quintile 4 | 720 (20) | 42,043 (18) |
| Quintile 5 (less disadvantage) | 826 (23) | 56,460 (24) |
| **Rx-Risk-V comorbidity category, n (%)** |  |  |
| 0-1 | 774 (12) | 16,857 (7) |
| 2-3 | 1,238 (18) | 42,269 (18) |
| 4-5 | 1,797 (27) | 66,633 (29) |
| 6-8 | 2,159 (32) | 80,242 (34) |
| ≥9 | 747 (11) | 27,566 (12) |
| **Number of unique medications in previous 90-days, n (%)** |  |  |
| 0-4 | 2,103 (31) | 63,407 (27) |
| 5-10 | 3,229 (48) | 120,045 (51) |
| ≥11 | 1,383 (21) | 50,115 (22) |
| **Antipsychotic use in previous 90-days, n (%)** | 417 (6) | 14,841 (6) |
| **High sedative load in previous year, n (%)** | 2,401 (36) | 82,549 (35) |
| **Chronic opioid use in previous year, n (%)** | 923 (14) | 30,369 (13) |
| **Health conditions** |  |  |
| Dementia, n (%) | 559 (16) | 47,028 (20) |
| History of falls, n (%) | 372 (11) | 37,045 (16) |
| History of fractures, n (%) | 314 (9) | 23,585 (10) |
| History of pressure injury, n (%) | 101 (3) | 10,811 (5) |
| Incontinence, n (%) | 380 (11) | 31,870 (14) |
| History of delirium, n (%) | 20 (1) | 2,127 (1) |
| Epilepsy, n (%) | 196) | 13,364 (6) |
| Parkinson's disease, n (%) | 290 (8) | 17,679 (8) |
| Stroke/cerebrovascular disease, n (%) | 415 (12) | 34,908 (15) |
| Diabetes, n (%) | 870 (25) | 57,812 (25) |
| Cancer, n (%) | 507 (14) | 39,630 (17) |
| Ischaemic heart disease, n (%) | 616 (18) | 46,743 (20) |
| Blindness, n (%) | 82 (2) | 6,383 (3) |
| Deafness, n (%) | 290 (8) | 30,833 (13) |
| **General/medical practitioner attendances in previous year, n (%)** |  |  |
| 0 | 499 (7) | 9,523 (4) |
| 1-5 | 966 (14) | 33,615 (14) |
| 6-15 | 2,855 (43) | 107,354 (46) |
| ≥16 | 2,395 (36) | 83,075 (36) |
| **General/medical practitioner attendances after hours in previous year, n (%)** |  |  |
| 0 | 5,450 (81) | 188,836 (81) |
| 1 | 656 (10) | 24,873 (11) |
| 2-4 | 439 (7) | 14,421 (6) |
| ≥5 | 170 (3) | 5,437 (2) |
| **Urgent General practitioner attendances after hours in previous year, n (%)** |  |  |
| 0 | 6,066 (90) | 208,640 (89) |
| 1 | 449 (7) | 17,603 (8) |
| 2-4 | 177 (3) | 6,339 (3) |
| ≥5 | 23 (0) | 985 (0) |
| **Optometric services in previous year, n (%)** |  |  |
| 0 | 4,200 (63) | 152,051 (65) |
| 1 | 1,811 (27) | 59,522 (26) |
| 2-4 | 667 (10) | 20,585 (9) |
| ≥5 | 37 (1) | 1,409 (1) |
| **Geriatric medicine attendances in previous year, n (%)** |  |  |
| 0 | 6,358 (95) | 218,303 (94) |
| 1 | 214 (3) | 10,013 (4) |
| ≥2 | 143 (2) | 5,251 (2) |
| **General practitioner management plan in previous year, n (%)** | 3,691 (55) | 131,431 (56) |
| **Health assessment in previous year, n (%)** | 1,719 (26) | 67,478 (29) |
| **Medicines review in previous year, n (%)** | 344 (5) | 11,744 (5) |

Q1: 25^th^ percentile. Q3: 75^th^ percentile; ARIA: Accessibility/Remoteness Index of Australia; NSAF: National Screening and Assessment Form.

**Supplementary Table 9**: Long-term home care recipients receiving carer payments by whether they reported being a carer

| **Characteristics*** | **Reported being a carer, n = 15,509** | **Not a carer,**  **n = 94,641** | **Total,**  **n = 110,150** |
| --- | --- | --- | --- |
| Not received carer payment, n (%) | 8,899 (57) | 92,502 (98) | 101,401 (92) |
| Received carer payment, n (%) | 6,610 (43) | 2,139 (2) | 8,749 (8) |

*Data only available for care recipients who completed a NSAF

**Supplementary Table** **10:** Effect of one-year increase in date of receiving first long-term home care service on probability of having an informal carer by age, sex, and dementia status

| **Characteristics** | **Odds ratio (95% CI)*** | **P-value** |
| --- | --- | --- |
| **Age, years** |  | **0.01*** |
| 65 | 0.99 (0.97, 1.00) | 0.08 |
| 70 | 0.98 (0.97, 0.99) | 0.005 |
| 75 | 0.98 (0.97, 0.99) | <0.001 |
| 80 | 0.98 (0.97, 0.98) | <0.001 |
| 85 | 0.97 (0.96, 0.98) | <0.001 |
| 90 | 0.97 (0.96, 0.98) | <0.001 |
| **Sex** |  | **<0.001*** |
| Male | 0.99 (0.98, 1.00) | 0.12 |
| Female | 0.96 (0.95, 0.97) | <0.001 |
| **Dementia** |  | **<0.001*** |
| Yes | 1.00 (0.98, 1.02) | 0.91 |
| No | 0.95 (0.94, 0.95) | <0.001 |
| **CALD status** |  | **0.04*** |
| Yes | 0.96 (0.95, 0.97) | <0.001 |
| No | 0.94 (0.94, 0.95) | <0.001 |
| **Remoteness** |  | **0.08*** |
| Major cities | 0.95 (0.95, 0.96) | <0.001 |
| Inner regional | 0.95 (0.94, 0.95) | <0.001 |
| Outer regional | 0.95 (0.94, 0.96) | <0.001 |
| Remote or very remote | 0.99 (0.95, 1.02) | 0.47 |

CI: Confidence Interval. Odds ratios from logistic regression models with interaction terms between date of receiving first long-term home care and each characteristic.

*P-value for interaction with date of receiving first long-term home care.
